# Integrating computational fluid dynamics into organ-on-chip systems: a glioblastoma-centred design and validation framework

**DOI:** 10.3389/fbioe.2025.1716813

**Published:** 2026-01-22

**Authors:** Hooman Taleban, Xinzhong Li, Zulfiqur Ali, Karunakaran Kalesh, Jai Prakash, Tugba Bagci-Onder, Barbara Breznik

**Affiliations:** 1 Centre for Biodiscovery, SHLS Life Sciences, School of Health and Life Sciences, Teesside University, Middlesbrough, United Kingdom; 2 University of Cumbria, Carlisle, United Kingdom; 3 Engineered Therapeutics Group, Department of Advanced Organ Bioengineering and Therapeutics, Technical Medical Centre, University of Twente, Enschede, Netherlands; 4 Koç University School of Medicine, Istanbul, Türkiye; 5 Faculty of Chemistry and Chemical Engineering, University of Ljubljana, Ljubljana, Slovenia

**Keywords:** AI, computational fluid dynamics, glioblastoma, In silicosimulation, *in vitro* modelling, microfluidic perfusion, organ-on-chip, tumour microenvironment

## Abstract

Glioblastoma GBM: Glioblastoma multiforme (GBM) remains one of the most lethal and treatment-resistant brain cancers, driven in part by the complexity of its tumour microenvironment (TME). While organ-on-chip (OoC) platforms offer more physiologically relevant models than traditional 2D or static 3D systems, their design remains largely empirical, lacking predictive control over flow conditions, biochemical gradients, and mechanical cues. Computational Fluid Dynamics (CFD) has emerged as a powerful tool to enhance the design, precision, and biological fidelity of OoC platforms. This comprehensive review highlights current limitations in replicating GBM’s biological complexity and technical constraints in device fabrication and maintenance, mapping them to specific CFD strategies. It synthesises current strategies into a structured workflow for integrating CFD into the design, optimisation, and validation of microfluidic tumour models—bridging engineering precision with biological complexity. In addition, validation frameworks reported in the literature are highlighted and mapped onto GBM-on-chip applications have been recommended, drawing on widely recognised international standards for engineering validation and regulatory modelling practices. Finally, this review positions CFD as a core component of GBM-on-chip development and explores how its integration with AI-based optimisation can advance the creation of more predictive, scalable, and biologically relevant *in vitro* tumour models.

## 1 Introduction

Glioblastoma (GBM), previously known as glioblastoma multiforme is the most aggressive primary brain tumour in adults. It grows fast, spreads deep into the brain, and resists all standard treatments—surgery, temozolomide chemotherapy, and radiation. Even with intensive care, median survival remains only 11–21 months (Akay et al., 2018). This poor prognosis is largely attributed to the GBM’s highly complex and heterogeneous tumour microenvironment (TME), which contributes to disease progression and treatment resistance (Maity et al., 2024).

The TME in GBM is composed of a wide array of cellular and non-cellular components, including tumour cells, immune cells, glial cells, neurons, endothelial cells, and extracellular matrix (ECM)—all of which dynamically interact within a confined and evolving microenvironment. Astrocytes are glial cells that typically maintain balance and support in healthy brain tissue. Around GBM, they become dysfunctional and reactive, releasing signals that help tumour cells migrate and resist cell death (Chang et al., 2016). Accumulating evidence in the field of “cancer neuroscience” show that neurons can become entangled in the tumour’s network. Through direct metabolic exchanges with cancer cells, these neurons unintentionally fuel the tumour’s growth and drive its progression (Xiao et al., 2022). Meanwhile, endothelial cells and pericytes, which form the blood–brain barrier (BBB), attempt to maintain oxygen and nutrient supply through angiogenesis (the growth of new blood vessels). The BBB is a highly selective barrier that protects healthy brain tissue by restricting the passage of potentially harmful substances, including many therapeutic agents. However, the angiogenic process in GBM is chaotic, producing abnormal, leaky vessels that lead to heterogeneous perfusion and localised hypoxia (Rosińska and Gavard, 2021). The tumour also disrupts the BBB, allowing immune cells such as macrophages, T-cells, and microglia to enter the brain—and reprogramming them to support tumour progression (Broekman et al., 2018).

The ECM, extracellular matrix—a physical and biochemical scaffold that supports tumour structure and influences cell behaviour—adds another layer of complexity. The ECM in GBM is structurally and mechanically distinct from healthy brain tissue. It is stiffer and more disorganised, making the tumour more aggressive while also affecting how it responds to treatment (Khoonkari et al., 2022; Kondapaneni et al., 2024). Together, these components form a highly dynamic and treatment-resistant ecosystem. Recreating this dynamic *in vitro* is challenging but essential. Standard models like 2D monolayers and static 3D spheroids lack the complexity of real tumour environments and often fail to predict how treatments will work in patients (Garnique et al., 2024).

**FIGURE 1:**
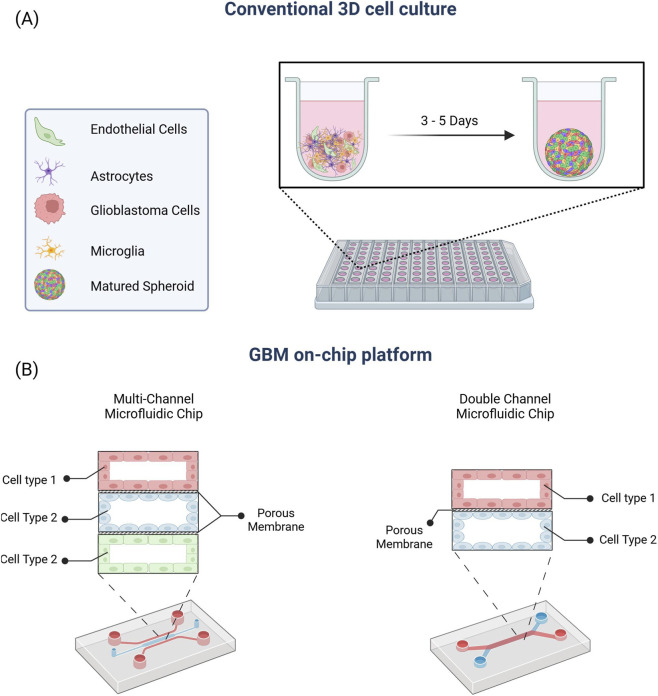
Composition of 3D GBM spheroids. **(A)** Schematic representation of co-culture of 3D GBM spheroids in 96 well plates. **(B)** OoC design featuring multiple microfluidic channels separated by porous membranes. This configuration allows individual cell types to be cultured under optimised conditions in separate channels, while enabling dynamic fluid flow and intercellular communication that mimic *in vivo* tissue interfaces. Created in BioRender. Taleban, H. (2025) https://BioRender.com/eae9pgw.

Organ-on-Chip (OoC) platforms, shown in Figure 1, offer promising alternative models. These systems integrate 3D cell cultures with precise control over microenvironmental conditions, incorporating tumour cells, brain-resident cell types, ECM elements, and nutrient dynamics in a scalable, tuneable setup (Ghamari et al., 2024; Maity et al., 2024). By mimicking *in vivo* features, GBM-on-chip models better replicate tumour behaviour, drug response, and disease progression. In fact, they can reveal therapeutic effects missed by conventional methods, including enhancing magnetic hyperthermia therapy (MHT) *via* improved nanoparticle delivery in GBM-on-chip systems (Mamani et al., 2020; Dorrigiv et al., 2023).

At the heart of OoC systems is microfluidics—the control of fluids at the microscale, often through channels just a few hundred microns wide. Microfluidic perfusion systems (MPSs) are devices developed to precisely control continuous flow of media, maintaining precise biochemical gradients, while removing waste (Tan et al., 2020). They enable dynamic observation of 3D cultures embedded in hydrogels or porous scaffolds (Meyer et al., 2023), supporting co-cultures of tumour cells with endothelial cells, astrocytes, and immune cells under dynamic flow to better simulate the BBB and assess drug permeability (Xiao et al., 2017; Straehla et al., 2021), and integrate biosensors for real-time monitoring of tumour-associated signals, which can provide insights into behaviours such as proliferation and migration (Thenuwara et al., 2024).

Numerical simulation, particularly Computational Fluid Dynamics (CFD), plays a vital role in designing and optimizing microfluidic devices. Experimental studies at the microscale can be time-consuming, expensive, and technically challenging, making computational modelling an attractive alternative or complement. Through numerical simulations, researchers can predict fluid behaviour, test different geometries, and optimise operational parameters, thereby reducing the reliance on multiple physical prototypes. CFD models are most powerful when validated against experimental data, enabling iterative refinement of both digital and physical models. CFD solves mathematical equations governing fluid behaviour, heat transfer, and species transport using methods like finite element, finite volume, or lattice Boltzmann approaches (Lax, 2007). These simulations help in understanding complex phenomena such as droplet formation, mixing, particle sorting, and cell manipulation under various forces like pressure, electric fields, or thermal gradients.

We review here the role of CFD in addressing GBM-on-chip limitations, with a focus on recent applications that enhance human relevance, design precision, and translational value.

## 2 Key challenges in GBM-on-chip platforms

Despite their demonstrated potential, GBM-on-chip platforms still face critical challenges for their broader applications in biomedical research and therapeutic development. Most of these challenges stem from the complex biology of GBM itself as well as the technical limitations of current microfluidic systems.

### 2.1 Biological and microenvironmental challenges

The GBM is diverse and variable at multiple levels, making it difficult to study and treat. This heterogeneity is important and replicating that *in vitro* remains a challenge for GBM-on-chip design.

#### 2.1.1 Replicating the TME and cellular heterogeneity

A major limitation in GBM-on-chip models is the oversimplification of cellular composition. Most models rely too heavily on GBM cells alone and overlook the immunological and stromal complexity of the native TME. These models primarily only incorporate GBM cells (Fan et al., 2016; Heinrich et al., 2019), neglecting the critical interactions with non-tumour cell populations such as immune cells, endothelial cells, neurons, and astrocytes. Some efforts have introduced endothelial cells alongside GBM cells (Xiao et al., 2019; Silvani et al., 2021), but these models still lack important supportive components such as pericytes and astrocytes. More advanced platforms incorporate multiple cell types, including GBM cells, endothelial cells, macrophages, and T-cells within a hyaluronan-based 3D matrix, thereby achieving higher physiological relevance (Cui et al., 2020). Omitting key subpopulations, such as therapy-resistant cells, can lead to misleading conclusions about treatment efficacy. The question is not whether models can replicate every component, but how much complexity is sufficient to be predictive of the tumour behaviour. CFD can help address this by simulating how missing cell types affect perfusion, nutrient gradients, and transport dynamics—offering insights into how reduced biological complexity may distort biophysical behaviour.

In addition, most GBM-on-chip studies rely on immortalised cell lines rather than patient-derived primary cultures, which limits their ability to reproduce patient-specific heterogeneity (Cui et al., 2020). An even greater challenge is reconstructing the TME from the same genetic background—for instance, ensuring that both the tumour cells and associated stromal or immune populations are derived from the same patient (Lessi et al., 2022). Without this alignment, key tumour–microenvironment interactions may be lost, reducing translational values.

#### 2.1.2 Incomplete replication of spatial heterogeneity

A second limitation lies in the misrepresentation of biochemical gradients. *In vivo*, GBM cell behaviour varies across the tumour due to steep gradients in oxygen, nutrients, and pH. GBM hypoxia plays a central role in shaping tumour biology. Low oxygen activates signalling pathways that promote invasion—searching for more oxygen-rich regions—and reduces the therapeutic efficacy (Pettersen et al., 2015; Richards et al., 2016). Blood flow is also chaotic in GBM, leading to uneven delivery of glucose and other nutrients. Cells near capillaries absorb most resources, while deeper regions become metabolically starved. This creates spatial diversity—cells at the edge might divide rapidly, while those in the core slow down or shift into survival mode (Ayuso et al., 2017; Thenuwara et al., 2024). Acidification further shapes the TME. High glycolytic activity generates lactic and carbonic acids, lowering extracellular pH—particularly near the hypoxic core (Miranda-Goncalves et al., 2016). This acidification helps the tumour evade the immune system, invade surrounding tissue, and withstand treatment (Thenuwara et al., 2024). These gradients shape tumour behaviour—driving invasion, adaptation, and therapy resistance.

Most chip-based models, however, rely on constant perfusion, flattening these gradients entirely. Uniform media flow ensures equal oxygen and glucose delivery, erasing metabolic differences and creating artificial homogeneity (Tripathy et al., 2024). Similarly, pH is stabilised system-wide, failing to recreate the natural acid–base variation between vascular and necrotic zones (Tajeddin et al., 2021). In actual tumours, hypoxic zones can emerge just 100 µm from the nearest blood supply (Bouquerel et al., 2023), but many microfluidic systems lack the spatial control to reproduce such steep local gradients (Ayensa-Jiménez et al., 2019). Accurate modelling of these gradients remains a challenge which is essential for physiological relevance. One complementary approach is the use of hypoxic incubators or gas-controlled chambers, which allow GBM cells to be cultured under defined oxygen tensions (e.g., 1% O_2_), revealing hypoxia-driven changes in gene expression, invasion, and treatment response (Macharia et al., 2021). Similarly, microfluidic systems have been developed to integrate oxygen control with perfusion, reproducing simultaneous hypoxic and flow conditions (Takahashi et al., 2023). Combining such experimental strategies with CFD-based modelling may enable both global and localised hypoxic gradients to be recreated more faithfully, enhancing the physiological relevance of GBM-on-chip models.

#### 2.1.3 Recreating the ECM dynamic characteristics

Matrix properties are another source of mismatch. The real brain’s ECM is soft, hyaluronic-acid (HA) rich, and dynamic. This matrix is constantly being remodelled by tumour and surrounding stromal cells, which deposit new components such as collagen, degraded matrix *via* enzymes such as matrix metalloproteinases (MMPs), and alteration of the mechanical stiffness over time (Xiao et al., 2017). These changes influence how cells migrate, sense mechanical cues, and respond to therapy. Additionally, since the real tumours are spatially heterogeneous, some regions are densely packed with ECM, while others are necrotic or fluid filled. Capturing this spatial variation in a single hydrogel formulation remains an open challenge (Bouquerel et al., 2023). Many OoC models use hydrogels composed of collagen I or Matrigel. While convenient, these materials differ from brain-specific ECM in stiffness, porosity, and viscoelasticity—differences that can significantly distort the physiological relevance (Kajtez et al., 2021; Khoonkari et al., 2022). To overcome this, researchers have begun developing HA-based hydrogels and nanocellulose-based materials that more closely mimic the brain ECM (dePalma et al., 2023; Ghamari et al., 2025). However, it remains difficult to fine-tune these materials to achieve the right balance between biochemical composition and mechanical stiffness. Moreover, most platforms still use a single, uniform ECM composition, overlooking the natural heterogeneity seen in real brain tumours.

#### 2.1.4 Recreating the blood–brain barrier’s heterogeneity

Finally, a key unresolved issue is the limited fidelity of BBB modelling. *In vivo*, brain endothelial cells experience stable, low shear stress (1–6 dyn/cm^2^) (Cucullo et al., 2011). This mechanical cue aligns the cells, supports polarisation, and drives the expression of proteins that seal the gaps between cells and regulate molecular transport. However, many microfluidic platforms struggle to establish or maintain these junctions, often due to the use of static or non-physiological flow profiles—whether excessively high, overly turbulent, or poorly defined.

In models that lack a BBB component, drugs are often applied directly to tumour cells. This setup bypasses the barrier entirely, leading to artificially high drug exposure. This may lead to false-positive results, overstating a drug’s therapeutic potential. Conversely, an overly strict endothelial barrier blocks drugs that would partially cross the compromised BBB seen in patients. The real challenge is not simply replicating a barrier, but mimicking its heterogeneous permeability—since in GBM, the BBB is not uniformly disrupted (Sarkaria et al., 2018; Conq et al., 2024). Models that reproduce this graded permeability may offer better predictions than binary “sealed or leaky” designs. Addressing BBB heterogeneity therefore remains one of the most critical steps in improving the physiological relevance of GBM-on-chip models, ensuring that drug screening outcomes more accurately reflect *in vivo* conditions.

### 2.2 Technical and operational limitations

Many GBM-on-chip systems struggle with engineering flaws that undercut their stability, precision, and reproducibility—qualities that are non-negotiable for reliable tumour modelling. These issues usually come from material choices, fragile system designs, and weak control over dynamic culture conditions. While not biological in nature, these technical gaps still prevent the models from accurately mimicking the TME.

#### 2.2.1 Material constraints and absorption artifacts

Polydimethylsiloxane (PDMS), the most widely used material for microfluidic prototyping, presents several well-documented limitations. It readily absorbs small molecules, which can alter drug concentrations and disrupt gradient formation—issues reported in both early and recent studies (Carvalho et al., 2021; Grant et al., 2021). As detailed in Section 3.2, accurately quantifying this loss is non-trivial and requires coupling fluid dynamics with solid-phase diffusion models. PDMS is also inherently hydrophobic, which hinders cell adhesion and promotes bubble formation in microchannels. These effects can impair cell viability and flow stability unless mitigated by surface treatments such as plasma activation followed by ECM coatings. Moreover, PDMS is highly oxygen-permeable, complicating the establishment and maintenance of hypoxic conditions that are critical for modelling the GBM microenvironment. While solutions such as parylene coatings or gas-permeable membranes with pre-equilibrated media have been proposed (Forry and Locascio, 2011), they only partially address the issue. Despite broad recognition of these drawbacks, most GBM-on-chip studies continue to rely on PDMS, favouring ease of fabrication over biological accuracy. While PDMS absorption is a well-known experimental limitation, most current CFD studies assume perfectly inert channel walls. This highlights a gap that Section 3.3 discusses in more detail, where modelling mass-transfer losses could improve drug-dose predictability.

#### 2.2.2 Environmental drift during long-term culture

Long-term experiments are vulnerable to environmental drift—gradual changes in temperature, pH, and flow rate that can skew biological outcomes and compromise model reliability (Tanyeri and Tay, 2018; Virtuoso et al., 2021). As an example, pump instability may cause a slow decline in flow rate, altering shear stress and nutrient gradients across the culture. Tubing materials may swell, collapse, or develop micro-blockages, further affecting perfusion.

Temperature and CO_2_ fluctuations can impact cell behaviour. GBM cells and their supporting populations require stable conditions of 37 °C with regulated oxygen and CO_2_ levels. Even a brief temperature increase can affect cell metabolism or stress responses (Abdulghany, 2025). Although OoC chips are typically kept in humidified incubators, imaging or sampling often requires removal, exposing systems to ambient conditions. While advanced live-cell microscopes are equipped with humidified chambers that maintain CO_2_, temperature, and humidity, not all systems have this capability, which can affect reproducibility. These fluctuations reduce assay repeatability and complicate data interpretation.

The pH is another factor since GBM is acidic and its high glycolytic activity generates lactic and carbonic acids that lower the extracellular pH, especially near the core of the tumour (Miranda-Goncalves et al., 2016). This acidification helps the tumour evade the immune system, invade surrounding tissue, and withstand treatment (dePalma et al., 2023). Therefore, maintaining this acidic environment *in vitro* is essential, although there is a trade-off to be made. Sealed systems allow acid buildup, but risk uncontrolled pH drops, which can damage cells (Ibrahim-Hashim and Estrella, 2019). Conversely, continuous perfusion maintains neutral pH, preventing the formation of an acidic core. Achieving an accurate pH gradient requires carefully balancing metabolic waste accumulation with controlled flow.

Sterility is equally important since a single contamination event in a microscale device can compromise the entire culture. The small volume and enclosed geometry prevent localizing or isolating the affected area. Warm, nutrient-rich environments are ideal for microbial growth, making weeks-long experiments especially vulnerable (Brueckner et al., 2017). Ensuring sterility over extended durations—while permitting sampling, media exchange, and gas exchange—remains a major logistical hurdle. Subtle mechanical failures often remain undetected until they affect the biological outcomes.

CFD typically assumes stable boundary conditions; however, real systems experience flow and temperature drift. Section 3 discusses how transient simulations can partially capture this behaviour.

#### 2.2.3 Perfusion instability and evaporation effects

Maintaining viable GBM cultures under dynamic flow conditions remains a major challenge in microfluidic platforms, particularly without integrated feedback or adaptive control (Barbosa L. C. et al., 2024). To do so, advanced OoC platforms increasingly integrate real-time sensors for tracking environmental conditions—oxygen tension (hypoxia), pH, temperature, and flow (Mughal et al., 2022). However, sensor drift, misalignment, or poor calibration can introduce significant measurement errors. For instance, oxygen sensors that consume trace amounts of O_2_ can slowly distort the very gradients they are meant to track. These errors can lead to false assumptions about local microenvironmental conditions, ultimately compromising data reliability.

Moreover, the microliter-scale volumes typical of these platforms are highly sensitive to evaporation, especially through PDMS. Even minor fluid loss can raise osmolarity, shift pH, and concentrate solutes, inducing metabolic stress or cell death (Heo et al., 2007; Forry and Locascio, 2011; Tanyeri and Tay, 2018). Evaporation also causes volume loss and air ingress, leading to bubble generation that disrupt flow, detach cell layers, and interfere with inline sensing (Kadam et al., 2021; Priy et al., 2024). To counter this, a fully humidified environment—or periodically topping up medium—is essential for maintaining long-term culture stability. While experimental mitigation is standard, predicting air entrainment risks requires advanced multiphase flow solvers (e.g., Volume of Fluid), as discussed in Section 3.2.

Bubble intrusion is rarely modeled directly in GBM-on-chip CFD studies due to the additional complexity of multiphase solvers. Section 3.2 outlines how standard laminar-flow models can still be used to identify regions prone to bubble entrapment.

### 2.3 Throughput and data collection constraints

Most OoC systems, including GBM models, are low-throughput by nature—typically handling one patient sample or condition per chip (Xie et al., 2023). Each device typically requires custom setup and skilled handling, which limits experimental throughput compared to conventional well-plates. Scaling up GBM-on-chip platforms for high-throughput applications presents both engineering and biological challenges. Expanding from a single microfluidic tumour chamber to dozens or hundreds demands precise control over flow conditions across all units. Even slight differences in resistance, geometry, or inlet design can lead to uneven perfusion, impacting outcomes.

Additionally, experiments often yield only minute volumes of fluid and a small number of cells, complicating analytical readouts (Ustun et al., 2021). Recent innovations are beginning to address these issues. Platforms integrating programmable fluid control and embedded biosensors now support up to 96 devices per plate (Azizgolshani et al., 2021). These sensors can monitor cell behaviour, barrier integrity, and molecular readouts in real time—key for dynamic drug assays (Mughal et al., 2022). However, real-time imaging inside microfluidic devices can still be complicated by optical limitations, material opacity, or complex channel geometries.

Quantifying fluid dynamics and transport remains a challenge. Key variables such as flow rate, shear stress, nutrient gradients, and oxygen distribution are often poorly defined or estimated using generalised assumptions (Barbosa F. et al., 2024; Carvalho et al., 2024). Yet even minor changes in shear stress can affect cell morphology and function, and many platforms lack tools for precise measurement (Candarlioglu et al., 2022; Espina et al., 2023). Techniques such as micro-particle image velocimetry (µPIV) or fluorescence-based imaging require complex setups and can disturb the conditions they aim to measure (da Ponte et al., 2021; Pisapia et al., 2022), while offering limited spatial and temporal resolution (Kingsmore et al., 2018; Tran et al., 2018).

Together, these limitations show that scaling up GBM-on-chip platforms requires not only parallelised designs but also reliable methods for quantifying microenvironmental conditions.

## 3 Role and importance of CFD in GBM-on-chip

CFD is a powerful tool for simulating microfluidic behaviour by solving the incompressible Navier–Stokes (N–S) equations (Equations 1, 2) under defined boundary conditions, which describe mass and momentum conservation within GBM-on-chip devices.

∇·u=0 (1)

$$\rho \;\left(\frac{\partial u}{\partial t}+u\cdot \nabla u\;\right)=-\nabla p+\mu \nabla^{2}u+f$$ (2)

Where u is the velocity field, representing how fluid moves in space; *ρ* is the fluid density (mass per unit volume); *p* is the pressure field, which drives the fluid through the microchannels; *μ* is the dynamic viscosity, indicating how resistant the fluid is to flow; ∇ (del operator) represents spatial gradients (changes over space) and f represents external body forces. Because microfluidic channels are so small, viscous forces (friction) dominate over inertial forces (momentum). Mathematically, this means the Reynolds Number is very low (Re <<1), making flow strictly laminar—ideal for describing perfusion through microchannels and along engineered vascular structures. In microfluidic systems, we can cross out the left side of the Equation 2, leaving us with the Equation 3, the linear Stokes Equation (García et al., 2022; Takken and Wille, 2022):

$$0=-\nabla p+\mu \nabla^{2}u+f$$ (3)

Modern CFD solvers use numerical schemes such as the finite volume or finite element method and, when needed, high-performance computing to obtain accurate solutions to these coupled partial differential equations (Pirouz et al., 2025). CFD therefore serves two complementary roles: (1) modelling the microenvironmental conditions that emerge inside OoC platforms (Chen et al., 2019; Hamad et al., 2021; Slay et al., 2024), and (2) predicting the effect of geometric or operational changes *in silico* before fabrication (Lichtenberg et al., 2020). Leveraging these two roles together is essential for improving the physiological fidelity and reproducibility of GBM-on-chip systems.

### 3.1 Addressing microenvironmental limitations

The potential use of CFD range from modelling flow distribution and convection–diffusion to shear stress profiling, mass transport simulation, and fluid–structure interaction (FSI). These can be used to overcome several of the biological and microenvironmental limitations outlined earlier. An overview of the governing equations, typical parameter ranges, and numerical assumptions used across GBM-on-chip CFD studies is summarised in Table 1.

**TABLE 1:** Mathematical models, governing equations, assumptions, typical parameter ranges, and numerical implementations used in CFD-based GBM-on-chip studies.

Model type	Governing equations	Assumptions	Boundary conditions	Typical parameter ranges (from literature)	Use in GBM-on-chip	Key studies
Incompressible laminar flow (N–S)	∇.u=0 $\rho \;\left(u.\nabla \right)=-\nabla p+\mu \nabla^{2}u$	Newtonian; laminar; incompressible; steady or transient	Velocity inlet; zero-pressure outlet; no-slip walls	ρ = 1,000 kg/m^3^; μ = 0.7–1.2 mPa s; Re = 0.01–10	Perfusion, shear stress mapping	Llenas et al. (2021), Bathory and Stefanelli (2022), Xie et al. (2023), Luchini et al. (2025)
Non-Newtonian blood flow	N–S with viscosity law μ(γ̇) e.g., Carreau–Yasuda	Blood shear-thinning	Same + shear-dependent viscosity	μ_0_ ≈ 0.16 Pa s μ ≈ 0.0035 Pa s λ ≈ 3.3 s	Mimicking vascular abnormalities	Ibrahim (2022), Qayyum et al. (2023), Yasmin et al. (2024)
Convection–diffusion–reaction (nutrients, drugs, oxygen)	$\frac{\partial C}{\partial t}+u.\nabla C=D\nabla^{2}C-R_{\left(C\right)}$	Constant D; dilute species; uniform consumption rate	Concentration inlet; no-flux walls	D_o2_ ≈ 2–3 × 10^-9^ m^2^/s D_drug_ ≈ 0.5–1.5 × 10^−10^ m^2^/s V_max_ ≈ 1–10 × 10^−3^ mol/m^3^·s	Oxygen gradients, drug penetration	Grant et al. (2021), Botte et al. (2023)
First-order cellular consumption	R = kC	Reaction-dominated regions	Zero-flux at walls	k ≈ 0.001–0.02 s^-1^	Early uptake models	Botte et al. (2023)
Michaelis–Menten kinetics	R=Vmax.CKm+C	Saturation at high C	Concentration-specified inlet	V_max_ ≈ 1–10 μmol m^-3^·s^-1^ K_m_ ≈ 0.1–1 μM	Realistic oxygen/drug consumption	Weilandt and Hatzimanikatis (2018), Hou et al. (2020)
Porous media flow (Darcy)	$\nabla p=-\frac{\mu }{K}u$	No shear; homogeneous K	Pressure drop BCs	K ≈ 10^−11^–10^−9^ m^2^ (hydrogels)	Hydrogel/interstitial flow	Zhu and Ma (2018), Elkady et al. (2022), Konopka and Carvalho (2025)
Porous media (Brinkman)	$\nabla p=\mu_{eff}\nabla^{2}u-\frac{\mu }{K}u$	Includes shear in gel	Fluid–porous interface	K ≈ 10^−12^–10^−9^ m^2^ μ as above	Gel–microchannel coupling	Zhu and Ma (2018), Elkady et al. (2022), Konopka and Carvalho (2025)
Chemotaxis/cell migration	$\frac{\partial n}{\partial t}=D_{n}\nabla^{2}n-x\nabla .\left(n\nabla C\right)+S_{\left(n\right)}$	Cells respond to gradients; no-slip at walls	Zero flux	χ ≈ 10^−8^–10^−7^ m^2^/mol D_n_ ≈ 10^−13^–10^−12^ m^2^/s	Hypoxia-driven invasion	Ibrahim (2022), Botte et al. (2023)
PDMS absorption/Wall permeability	$J=P\left(C_{f}-C_{w}\right)$	Wall acts as sink; partitioning	Flux continuity	P ≈ 10^−5^–10^−3^ m/s KP/F ≈ 2–20	Drug loss; O_2_ ingress	Shirure and George (2017), Grant et al. (2021)
VOF/level set multiphase flow	$\frac{\partial \alpha }{\partial t}+\nabla .\left(\alpha u\right)=0$	Immiscible phases; explicit surface tension	Inlet gas/liquid mix	σ ≈ 0.07–0.075 N/m (air–water)	Bubble formation, trapping	Zhu and Ma (2018), Carrillo and Bourg (2021)
Thermal transport	$\rho C_{p}=k\nabla^{2}T$	Negligible convection	Fixed T or convective BC	*k* ≈ 0.6 W/m·K (water)	Temperature uniformity	Peng et al. (2019), Zhao et al. (2021), Munoz et al. (2024)
FSI	N–S + solid mechanics: $\sigma_{s}+f=0$	Elastic deformation; quasi-steady	Coupled wall-fluid	E ≈ 1–10^5^ Pa (hydrogels)	ECM deformation, vessel pulsatility	Menon et al. (2014), Wang et al. (2023)

#### 3.1.1 Cellular and TME complexity

CFD provides a quantitative framework for examining how the absence or inclusion of key non-tumour cell populations impacts local flow dynamics and nutrient distribution. Cellular reaction kinetics, such as oxygen consumption, can be incorporated directly through a depletion term in the Equation 4 (advection–diffusion):

$$\frac{\partial C}{\partial t}+u.\nabla C=D\nabla^{2}C‐R_{\left(C\right)}$$ (4)

where C_(x,t)_ is the solute concentration, u is the fluid velocity obtained from N–S solutions, D is the diffusion coefficient, and R_(C)_ represents cellular uptake. In practice, R_(C)_ is often modelled using either first-order kinetics $R_{\left(C\right)}=kC$ , or Michaelis–Menten kinetics $R_{\left(C\right)}=\frac{V_{\max}\;.\;C}{K_{m}+C}$ , depending on whether saturation effects need to be captured (de Montigny et al., 2021). Here, *k* is the first-order rate constant, *V*_max_ is the maximum reaction velocity, and *K*_*m*_ is the Michaelis constant, which is the substrate concentration at which the reaction rate is half of *V*_max_.

Conventional CFD frameworks typically treat the tumour region as a continuous, homogeneous sink. Cellular activity and spatial heterogeneity are averaged into a single continuum term (Sweeney et al., 2019). This approximation is computationally efficient, but it smooths out the inherently heterogeneous and stochastic behaviour of individual cells and clusters (Hormuth et al., 2021). To overcome this limitation, more advanced hybrid frameworks couple discrete cell representations—using agent-based models or discrete phase models—with the continuum CFD fields (Mmereke et al., 2025). In these multiscale approaches, individual cells can act as discrete sinks and physical obstacles to flow, so that local nutrient micro-gradients form around clusters and interfaces. This level of description captures effects that a purely continuum model cannot (Norton et al., 2019). For micro-engineered tumour-on-chip systems, such as GBM analogues, physiologically meaningful predictions increasingly depend on moving beyond a uniform sink description and adopting hybrid or multiscale models that preserve local inhomogeneity, cell–cell contact, and cell–matrix compaction.

Such *in silico* experiments make it possible to systematically probe how specific cell populations—astrocytes, immune cells, endothelial components—reshape flow fields and nutrient landscapes (Palacio-Castañeda et al., 2021; Carvalho et al., 2025). For instance, if removing a stromal compartment in the model produces unrealistically severe nutrient depletion compared with experimental data, this discrepancy may indicate the need to re-introduce that cell type or to adjust the feeding regime (for example, by changing seeding density or perfusion rate). In this way, CFD-guided analysis can inform the design of co-culture configurations, seeding strategies, and spatial arrangements in tumour-on-chip platforms, helping them better reproduce in vivo-like oxygen and nutrient gradients and the associated patterns of hypoxia and necrosis.

#### 3.1.2 Spatial heterogeneity and gradient formation

By solving convection-diffusion equations (often coupled with cell consumption kinetics), CFD predicts nutrient concentration profiles across the microfluidic chip. For example, it has been used to replicate perivascular oxygenation patterns, characteristic of hypoxic tumour cores—enabling fine-tuning of flow rates and channel geometry for greater physiological relevance (Carvalho et al., 2021). Convection–diffusion simulations have also revealed that even under uniform perfusion, spatial heterogeneity in oxygen and nutrient availability persists, revealing complexities missed by simplified models (Naşcu et al., 2022). CFD also quantifies mechanical cues such as local shear stress τ experienced by cells (Lo et al., 2013). showed that varying shear stress modulates GBM cell morphology and gene expression. Simulations allow precise mapping of shear stress across the culture surface by adjusting flow rate, inlet/outlet designs, or introducing obstacles. This level of control helps recreate a biochemical environment that more closely resembles the conditions within an actual tumour.

To accurately predict biochemical gradients in GBM-on-chip platforms, it is critical to distinguish between stationary (steady-state) and transient (time-dependent) numerical formulations—a distinction often overlooked in the biological literature. While microfluidic flow is typically laminar (*Re*≪1) and often modelled as steady-state using the N–S equations, solute transport processes are frequently time-dependent (Carvalho et al., 2024). Most GBM-on-chip studies use Equation 5 (stationary convection–diffusion formulation) to estimate equilibrium nutrient distributions:

$$\nabla .\;\left(-D\nabla c+uc\right)=R$$ (5)

Where *D* is the diffusion coefficient, u is the velocity field, and *R* represents the reaction term (metabolic consumption). While sufficient for predicting baseline oxygenation in long-term culture, this formulation fails to capture dynamic phenomena essential to GBM treatment, such as drug pulsing or the temporal establishment of gradients (Ayensa-Jiménez et al., 2020; Menezes et al., 2024). To simulate such dynamics, transient formulations must include an accumulation term. The result is Equation 6 (unsteady convection–diffusion):

$$\frac{\partial c}{\partial t}+\nabla .\left(-D\nabla c+uc\right)=R$$ (6)

Transient models are computationally more expensive but are strictly necessary for simulating pharmacokinetic profiles (e.g., the wash-in and wash-out of a drug) or the time-lag in oxygen depletion following a flow interruption (Regmi et al., 2022). A significant limitation in current GBM-on-chip literature is the reliance on steady-state assumptions when evaluating drug efficacy, neglecting the time-dependent exposure patterns cells experience *in vivo* (Logun et al., 2018).

Ultimately, nutrient gradients and mechanical stresses naturally emerge even under nominally uniform flow conditions. CFD provides a quantitative framework for mapping these spatiotemporal variations and for rationally designing microfluidic architectures that more faithfully recapitulate the biochemical and mechanical microenvironment of GBM tumors.

#### 3.1.3 ECM composition and mechanical cues

Conventional CFD fails to capture the dynamic properties of the brain ECM. It assumes static, rigid geometries and cannot explain how soft and porous tissues like the ECM deform when fluid flows through them. This limits its ability to simulate solute transport or mechanical feedback in soft matrices.

To address this, there is increasing interest in integrating CFD with FSI simulations—coupling fluid dynamics with structural mechanics simulations (e.g., finite element methods). This offers a two-way approach that models both fluid flow and the deformation of soft materials like the ECM under flow. In an FSI model, the fluid phase follows the N–S equations, while the solid phase is described using continuum mechanics—often linear elasticity for small deformations. At the fluid–solid interface, the model enforces continuity of velocity and stress: fluid shear stresses must balance solid stresses, and the no-slip condition applies even on moving boundaries. Although computationally demanding, this approach reveals how soft hydrogels respond to sustained shear and pressure. Notably (Cherubini et al., 2023), showed in a fetoplacental microvessel-on-chip that sustained shear stress stiffened the surrounding matrix and reduced solute diffusivity, effectively turning the ECM into a denser transport barrier. Extending this FSI framework to GBM-on-chip platforms, will allow exploration of how properties such as matrix stiffness and compliance affect local shear stress, pressure distribution, and diffusive gradients (Musharaf et al., 2024). This is especially important in systems using soft, brain-like hydrogels. When material properties are well characterised, FSI models offer a useful approximation of how structural properties shape the biochemical landscape.

When fully coupled FSI models become impractical, porous-media approximations offer a useful alternative. In this approach, soft hydrogels are treated as porous continua whose hydraulic resistance is governed by the permeability κ and the effective viscosity *μ*_*eff*_. The resulting Equation 7 (Brinkman momentum) is commonly expressed as:

$$\nabla p=\mu_{eff}\;\nabla^{2}u-\frac{\mu }{\kappa }u$$ (7)

For comparison, the classical Darcy formulation (Equation 8) can be written as:

$$\nabla p=-\frac{\mu }{K}u$$ (8)

Here, *κ* (often used interchangeably with K) reflects hydraulic permeability of the hydrogel (derived from pore size) and *μ*_*eff*_ is the effective viscosity of fluid flow through the pore network. Because the Brinkman model retains the viscous dissipation term ( $\mu_{eff}\;\nabla^{2}u$ ), it is more appropriate than Darcy’s law for intermediate-porosity hydrogels commonly used in GBM-on-chip platforms (Esposito et al., 2022; Del Mastro et al., 2025).

Permeability values (10^−14^–10^−12^ m^2^) define resistance to interstitial flow, assuming a static domain with constant permeability. However, this simplification is a significant limitation for GBM modelling. In biological reality, GBM cells actively remodel the ECM *via* Matrix Metalloproteinases (MMPs), changing the local porosity and permeability over time. Current CFD models largely fail to couple this biological remodelling with fluid dynamics, relying instead on fixed parameterisations of κ that do not evolve during the simulation. Incorporating poroelastic frameworks or FSI models that account for this bidirectional coupling would significantly enhance the predictive power of GBM-on-chip simulations (Yuan et al., 2023).

In both cases, calibrating model parameters (Young’s modulus, hydraulic permeability, *etc.*) against experimental measurements is essential. Overall, these approaches broaden the scope of CFD from pure fluid behaviour to biophysical interactions at the tissue level.

#### 3.1.4 BBB fidelity

Recreating the BBB *in vitro* demands tight control over wall shear stress (WSS), the key mechanical cue that regulates endothelial alignment and tight-junction formation. In CFD, WSS (*τ*_*w*_) is obtained from the local velocity gradient at the endothelial wall, described by Equation 9:

$$\tau_{w}={\mu \left(\frac{\partial u}{\partial y}\right)}_{y=0}$$ (9)

where *μ* is the dynamic viscosity and *u* is the tangential velocity. Physiological WSS in brain microvessels typically falls within 1–10 dyn/cm^2^, a range known to promote endothelial quiescence and barrier maturation (Noorani et al., 2021). Many CFD studies simplify the perfusate as a Newtonian fluid. Blood, however, is non-Newtonian and exhibits shear-thinning behaviour that directly influences local viscosity and shear stress, especially near vessel walls. Constitutive laws such as the Carreau–Yasuda or power-law models better capture these effects and provide more accurate WSS predictions in low-shear regions (Ceccarelli et al., 2024). Non-Newtonian modelling can reduce WSS error by more than 40% in these zones, underscoring its importance in BBB and cerebrovascular chip design (Noorani et al., 2021).

Permeability is the second major factor in BBB modelling. It governs solute exchange between vascular and parenchymal compartments. In most advection–diffusion CFD formulations, the BBB is represented not as a discrete physical layer but as a flux boundary condition, described by Equation 10:

$$J=P_{app}\;\left(C_{vascular}-C_{tissue}\right)$$ (10)

where *J* is the solute flux and *P*_*app*_ is the apparent permeability coefficient. GBM- and BBB-on-chip models often treat *P*_*app*_ as a uniform constant, typically measured through tracer permeability assays. This assumption neglects the highly heterogeneous permeability of glioblastoma vasculature, where relatively intact vessels exist alongside leaky, tumour-associated endothelium (Ceccarelli et al., 2024). More realistic simulations require spatially variable permeability fields—models in which P_app_ depends on local hemodynamics or structural cues, such as shear stress or tumour proximity. Such formulations better capture region-to-region variability in drug penetration and efflux across a disrupted BBB.

Therefore, tuning the flow rate and channel geometry can allow the recreation of the required physiological shear stress range which is essential for proper endothelial function (Fan et al., 2023). Numerical simulations enable the exploration of how vascular architecture and barrier permeability influence drug distribution into brain tissue, offering a predictive tool for optimizing chip design (Hassanzadeganroudsari et al., 2020; Gkountas et al., 2021).

It is important to note that CFD can point to ideal flow conditions and forecast transport dynamics, but these predictions need experimental confirmation (Ahmed et al., 2021). Cell behaviour, especially in response to shear stress and microenvironmental cues, varies widely depending on the context.

### 3.2 Mitigating technical and operational constraints

CFD offers a powerful way to predict—and ultimately mitigate—the material and operational challenges that arise in microfluidic tumour models. Instead of relying on trial-and-error fabrication, CFD allows researchers to evaluate solute loss, gas permeability, bubble formation, and flow instability quantitatively, turning device development into a more systematic engineering process.

#### 3.2.1 Material interactions (PDMS absorption)

Drug loss and oxygen leakage through PDMS can be captured by adding diffusion in solid domains and appropriate interface conditions. At the fluid–PDMS boundary, solute transfer can be approximated by a flux driven by the concentration gradient represented by Equation 11:

$$J=k\;\left(C_{fluid}-C_{wall}\right)$$ (11)

where *J* is the mass flux, *k* is the mass-transfer coefficient, and *C* denotes solute concentrations in the fluid and wall. A more rigorous method solves Fick’s second law in both the fluid and PDMS regions, linking them through a partition coefficient (*K*_*P/F*_) that defines equilibrium between the two phases (Equation 12):

$$J=K_{P/F}\;C_{fluid}$$ (12)

This is coupled with Equation 13 (transient solid-phase diffusion) within the device walls:

$$\frac{\partial C}{\partial t}=D_{PDMS}\nabla^{2}C$$ (13)

where *D*_*PDMS*_ is the solute diffusion coefficient within PDMS. This coupled formulation allows time-dependent prediction of drug loss or oxygen ingress across PDMS walls under realistic culture conditions (Shirure and George, 2017). Using experimentally measured diffusion and partition coefficients—reported for compounds such as paclitaxel (Grant et al., 2021; Barbosa F. et al., 2024)—one can quantify how much the effective exposure inside the device deviates from the intended dose. To capture oxygen transport, Henry’s law constants can be implemented at the PDMS–fluid interface, enabling simulation of gas flux across permeable walls. This is essential when reproducing hypoxic tumour microenvironments, where even minimal leakage can disrupt oxygen gradients.

These results then inform material choices, for example, switching to cyclic olefin polymers or glass, or using surface coatings to reduce absorption. Similarly, simulations can test the impact of adding an oxygen-impermeable layer (e.g., PMMA) and compare oxygen profiles with and without this barrier, ensuring that a designed hypoxic region remains hypoxic.

#### 3.2.2 Flow stability and bubble formation

Transient CFD captures flow oscillations from peristaltic or syringe pumps, as well as deformation-induced variations in shear stress or nutrient delivery.

Single-phase CFD can identify recirculation zones, stagnation points, and sudden expansions prone to bubble entrapment, while multiphase models—such as the Volume of Fluid (VOF) or Eulerian–Eulerian methods—explicitly track air–liquid interfaces. These methods solve a transport equation such as Equation 14 for the phase volume fraction (*α*):

$$\frac{\partial \alpha }{\partial t}+\nabla .\;\left(u\alpha \right)=0$$ (14)

where *α* = 1 represents the fluid and *α* = 0 represents the gas (or *vice versa*). This allows for the visualization of bubble trajectories, preferred flow paths, and trapping sites (Kadam et al., 2021). Transient models also support robustness analyses, introducing small perturbations in geometry, viscosity, or inlet pressure to assess design sensitivity. Systems that maintain stable flow and nutrient distribution under these variations are more reliable for long-term experiments. Recent transient multiphase simulations have further characterised slug-flow behavior, bubble velocity, and pressure oscillations in gas–liquid systems, improving predictive control of two-phase transport in microfluidic networks. Evaporation, particularly in open or semi-open culture systems, can be incorporated into these models by adding a phase-change mass source term (*ṁ*) to the continuity equation. This enables prediction of osmolarity shifts and nutrient concentration changes over time, informing the design of microfluidic platforms that sustain stable media composition and cell viability during long-term perfusion.

### 3.3 Enhancing throughput and data collection

A key strength of CFD lies in optimizing complex distribution networks to ensure uniform flow rates and pressures across multiple chambers. It has been used to successfully parallelise droplet-on-chip systems from one to thousands of units by identifying how to split flows evenly without interference (Aladese and Jeong, 2022). In the context of GBM, multi-chamber OoC devices culturing up to 128 patient-derived tumour samples under continuous perfusion have demonstrated the importance of uniform microenvironments—conditions that CFD can help design and validate (Olubajo et al., 2020).

Beyond scaling, CFD accelerates design iterations by reducing reliance on trial-and-error prototyping. *In silico* simulations save time, materials, and cost (Kou et al., 2011; Bakuova et al., 2023). This approach has improved flow uniformity, eliminated dead zones, and optimised mixing and drug delivery strategies (Aladese and Jeong, 2022; Khani et al., 2022; Mahmud, 2022). Emerging AI-driven CFD approaches further expand these capabilities. Techniques like deep reinforcement learning (e.g., Proximal Policy Optimisation) offer control over microfluidic droplet generation, enabling closed-loop feedback with high reliability (Gyimah et al., 2023). Similarly, multi-objective optimisation using machine learning (ML) and evolutionary algorithms has been shown to reduce mixing length and improve micromixer efficiency (Kouhkord et al., 2025). These AI-CFD hybrid methods streamline parameter tuning, reduce bias, and democratise simulation tools by automating setup and analysis (Ebner and Wille, 2023).

CFD also supports data collection. By modelling analyte transport, it can suggest optimal sensor placement for tracking chemical gradients or metabolic activity (Wong et al., 2017). It enables non-invasive estimation of fluid dynamics, pressure fields, shear stress, and solute distribution, helping to bypass the need for direct *in situ* measurements. Studies have mapped spatial variation in oxygen and drug delivery, revealing diffusion patterns and gradients undetectable with conventional imaging (Mulpuru et al., 2019; Komen et al., 2020). However, model accuracy depends on high-quality input data. Shear stress values, for example, are often calculated using simplified assumptions or generic flow profiles. Without calibration against experimental data, these predictions can be misleading.

As summarised in Table 2, various biological and technical challenges associated with GBM-on-chip platforms can be systematically addressed through specific CFD strategies, enabling more predictive and physiologically relevant models.

**TABLE 2:** Summary of key challenges in GBM-on-chip platforms and corresponding CFD strategies for addressing them. The table categorises limitations and challenges related to biological fidelity, material constraints, environmental stability, and data acquisition, and links each to CFD capabilities such as flow simulation, shear stress profiling, multiphase modelling, and FSI.

​	Challenge (Section 2)	Description	CFD capability (Section 3)	How CFD helps
Biological and microenvironmental limitations	2.1. TME and cellular heterogeneity	Oversimplified cellular composition; lacks immune/stromal diversity	3.1. Multiphase CFD + reaction-diffusion models	Models impact of missing cell types on mechanical and biochemical gradients
	2.1. Spatial heterogeneity	Constant perfusion erases pH, nutrient, and O_2_ gradients	3.1. Convection-diffusion + metabolic consumption modelling	Simulates localised gradients; adjusts flow for physiological relevance
	2.1. ECM dynamics	Unrealistic ECM stiffness/composition	3.1. Fluid-Structure Interaction (FSI) modelling	Simulates ECM deformation and feedback
	2.1. BBB fidelity	Binary or absent BBB; lacks variable permeability	3.1. Shear stress profiling + barrier transport modelling	Tuning perfusion to mimic physiological shear and permeability
Technical limitations	2.2. PDMS absorption and permeability	Absorbs drugs, allows unintended O_2_ permeation	3.2. Modified boundary conditions and wall transport coefficients	Predicts solute loss and O_2_ diffusion through device walls
	2.2. Environmental drift	Fluctuations in flow, temp, pH*etc.*	3.2. Time-dependent CFD simulations	Forecasts impact of drift and guides environment control
	2.2. Perfusion instability and evaporation	Evaporation, flow loss, bubble formation	3.2. Multiphase flow + convection–diffusion modelling	Identifies high-risk zones for flow disruption and evaporation
Low Throughput and poor data collection	2.3. Low Throughput and poor quantification	Limited chip scaling, insufficient fluid data	3.3. Flow optimisation + parallel design simulation	Ensures uniform flow and supports chip scaling
	2.3. Poor data collection/resolution	Hard to track gradients or flows directly	3.3. In silico profiling of gradients, shear stress, velocity	Guides sensor placement and supplements low-res data

## 4 Strategic integration of CFD in GBM-on-chip development

**FIGURE 2:**
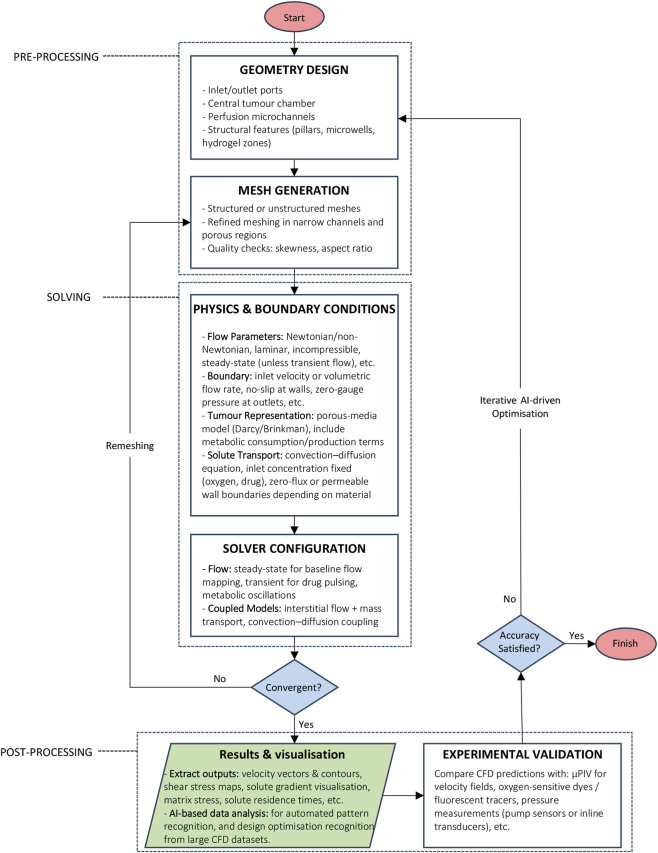
Standard CFD Workflow for GBM-on-chip Design Optimisation. The workflow is divided into three main stages: (1) Pre-processing (geometry definition and mesh generation), (2) simulation setup (solver and boundary condition specification), and (3) post-processing and validation (visualisation, parametric studies, and experimental confirmation). The process includes iterative optimisation of flow parameters, solute transport, and boundary conditions, with outputs validated using experimental data such as velocity fields (e.g., μPIV), oxygen-sensitive dyes, or pressure measurements. Using AI/ML algorithms accelerates parameter tuning, improves convergence speed, and enables closed-loop feedback for microfluidic control. This pipeline supports predictive modelling of flow behaviour, nutrient and drug gradients, and shear stress distribution in microfluidic platforms simulating the glioblastoma microenvironment.

Accurate CFD analysis of GBM-on-chip platforms requires more than solving the N–S equations. It demands a numerical workflow capable of capturing how perfusion, solute transport, porous hydrogels, and tumour-driven gradients interact across scales. The following sections outline a focused, engineering-oriented strategy for implementing CFD as a predictive modelling tool rather than a mere visualisation aid, as demonstrated in Figure 2.

### 4.1 Solver configuration and discretisation

Most microfluidic GBM models operate within the incompressible, laminar flow regime. Coupled solvers are generally preferable when the geometry includes porous regions or when pressure–velocity coupling becomes stiff, as they offer greater numerical stability than segregated approaches. Schemes such as SIMPLE or PISO remain efficient for simple channel flows, but they often struggle when applied to high-resistance or multiphysics domains common to tumour-on-chip devices (Giacomini et al., 2025).

Spatial discretisation must be handled with equal care. Second-order schemes are necessary to resolve steep nutrient and oxygen gradients. Upwind schemes, although robust, tend to introduce excessive numerical diffusion; higher-order formulations such as MUSCL or QUICK offer better accuracy when convection plays a significant role in solute transport (Kheiri et al., 2021; Giacomini et al., 2025). For long-duration simulations, fully implicit temporal discretisation provides stable convergence and avoids the timestep restrictions that would otherwise arise over biologically relevant timescales.

### 4.2 Mesh design and convergence assessment

Mesh quality is one of the strongest determinants of CFD accuracy in microfluidic systems. GBM-on-chip geometries—with cell-laden hydrogels, BBB-like channels, and narrow transition zones—require targeted mesh refinement near walls, interfaces, and geometric discontinuities (Ceccarelli et al., 2024; Yue et al., 2025). Inflation layers are particularly important for resolving near-wall shear stress, a variable central to endothelial alignment and barrier integrity (Grant et al., 2021).

Ensuring mesh independence is essential. Velocity, shear stress, and concentration fields should be compared across successive refinement levels, ideally converging within 2%–5% (Aycan et al., 2023). Reporting metrics such as minimum cell size, orthogonality, skewness, and convergence criteria strengthens reproducibility and allows readers to assess the reliability of the simulation (Wang et al., 2022).

### 4.3 Multiphysics coupling and timescale bridging

GBM-on-chip systems frequently incorporate porous hydrogels, spatially heterogeneous permeability, and dynamic microenvironmental changes. Porous tumour regions are most accurately represented using Brinkman or Darcy formulations, which enforce continuity of velocity and stress at the interface between free-flow and porous zones. This enables realistic modelling of interstitial flow and solute penetration through tumour-mimetic matrices (Carrillo et al., 2020).

Material interactions also require explicit treatment. Solute loss into PDMS walls, for example, should be modelled through partition-coefficient boundary conditions rather than by assuming impermeable walls—a common but often invalid simplification (Grant et al., 2021).

A persistent numerical challenge lies in bridging disparate timescales: fluid fields equilibrate in milliseconds, while biochemical or cellular processes unfold over hours or days. Fully transient simulations across biological periods are rarely feasible. A quasi-steady-state (QSS) workflow offers a practical alternative (Kheiri et al., 2021). Here, the flow field is solved under steady conditions, and species transport is computed on top of that fixed field. Biological parameters—consumption rates, porosity, ECM stiffness—are updated externally. The flow is recalculated only when structural or material changes exceed a predefined threshold. This strategy captures long-term evolution while keeping computational cost manageable.

### 4.4 Validation and verification

Predictive CFD requires rigorous numerical verification alongside experimental validation. Solver residuals, mass-balance errors, and sensitivity to relaxation parameters must be evaluated to confirm numerical stability and consistency.

These computational checks must be paired with experimental benchmarks. Techniques such as micro-Particle Image Velocimetry (*µ*PIV) for velocity fields, fluorescent dye tracing for solute transport, and oxygen or pH-sensitive probes for microenvironmental calibration provide essential ground truth (Etminan et al., 2022). Agreement between simulation and experiment is critical before using CFD to predict gradient formation, drug penetration, or shear environments within GBM constructs.

### 4.5 Barriers to broader adoption of CFD in GBM-on-chip research

Despite its value, several challenges limit the widespread use of CFD in OoC design applications. The primary limitation is computational cost. Realistic simulation of 3D TMEs—especially those modelling mass transport, non-Newtonian fluids, or cell–fluid interactions—typically require powerful workstations or access to high-performance computing clusters. These resources are not universally available, and very few models incorporate them in their models (Metzcar et al., 2019; Yang et al., 2023). Even with dedicated hardware, simulations may take days or weeks to complete (Takken and Wille, 2024). To address this issue, researchers often simplify models, for instance, by assuming 2D or steady-flow assumptions, or neglecting permeability, though such simplifications compromise the level of detail captured (Xie et al., 2023).

Expertise is another barrier since advanced CFD modelling involves mesh generation, parameter tuning, and often custom scripting—skills rarely found in biology-focused labs. While advanced commercial platforms like ANSYS^®^ Fluent and COMSOL Multiphysics® provide comprehensive toolsets, they remain costly and may be out of reach for smaller institutions. On the other hand, open-source packages such as OpenFOAM® are free but require steep technical knowledge. Their interfaces are less intuitive, and they often lack pre-built modules suited for microfluidic or biological applications, introducing additional burden to users (Carvalho et al., 2021; Ebner and Wille, 2023). Emerging AI, ML, and generative AI approaches offer promising ways to reduce these barriers. Surrogate and reduced-order models trained on CFD datasets can cut computational costs dramatically by predicting flow behaviour without rerunning full simulations (Kochkov et al., 2021). Generative AI tools can automate geometry generation, meshing, and parameter tuning, while reinforcement learning can assist in adaptive control of perfusion or gradient maintenance (Chen et al., 2024). Such methods also make CFD workflows more accessible to non-experts by embedding complex optimisation into user-friendly interfaces. By integrating these approaches with cloud-based simulation platforms, CFD may become a practical and scalable tool even in biology-focused laboratories with limited computational resources. Table 3 provides an overview of representative CFD-enabled studies, categorised by research objective and model type. This table summarises published studies applying CFD across four key domains of GBM-on-chip development: (1) drug delivery optimisation, (2) vascular modelling and biomechanics, (3) tumour microenvironment modelling, and (4) platform and device design for drug testing. For each study, the role of CFD, its specific contribution to GBM-on-chip design, and the associated experimental validation methods (if any) are listed. The table highlights the diversity of CFD applications in both modelling biological phenomena and improving device performance, as well as ongoing challenges in experimental validation and clinical translation.

**TABLE 3:** Representative applications of Computational Fluid Dynamics (CFD) in GBM-on-chip platforms, organised by research focus.

Main application focus	Role of CFD	Relevance to GBM-on-chip design	Validation method	References
Drug Delivery Optimisation	Simulates blood flow in vessels and models the fluid-particle interactions, especially how MNPs navigate and penetrate the BBB under various conditions.	Optimizing nanoparticle delivery strategies using magnetism, as a potential treatment for GBM.	Percentage of MNPs crossing the BBB Vs. experimental *in vitro* data from literature	Gkountas et al. (2021)
	Models blood flow in normal and tumour-mimicking capillaries to assess how vessel diameter and flow velocity influence nanoparticle transport and retention time. Uses particle trace analysis to quantify retention differences between healthy and tumour vasculature.	Replicating tumour vessel dilation and flow conditions to optimise nanoparticle-based drug delivery for GBM treatment.	CFD simulations were not directly validated experimentally (qualitatively validated against experimental cytotoxicity results)	Mulpuru et al. (2019)
	Implements a binding–diffusion mathematical model to simulate therapeutic protein (DARPin) diffusion, binding, and internalisation in both spheroids and microfluidic tumour-on-a-chip systems.	Guiding protein therapeutic delivery strategies in GBM-on-chip by predicting diffusion and binding dynamics, estimating minimal effective doses, and optimizing chip design to model realistic transport barriers.	Predicted DARPin penetration depth and concentration and diffusion profiles Vs. quantitative image-based experimental data	Palacio-Castañeda et al. (2021)
	Simulates the 2D and 3D mass transfer profiles of nanoparticles, quantifying concentration gradients and diffusion resistances across cellular and extracellular barriers.	Replicating realistic nanoparticle transport dynamics and identifying key physical parameters that influence drug delivery efficiency in GBM-on-chip platforms.	Simulated nanoparticle mass transfer resistance across the BBB Vs. experimental data from literature	Hassanzadeganroudsari et al. (2020)
	Simulates flow to predict velocity fields, pressure distribution, and shear stress on endothelial layers, enabling precise replication of physiological BBB conditions. Also models nutrient and solute transport to assess permeability under different flow rates and geometries.	Guiding microfluidic chip design to reproduce in vivo-like BBB shear stress and transport dynamics, ensuring realistic nutrient/drug delivery in GBM-on-chip systems.	Simulated tumour perfusion Vs. *in vivo* ASL-MRI perfusion measurements for the same tumours	Sweeney et al. (2019)
	Simulates fluid flow and heat transfer in vasculature-mimicking geometries, to identify optimal MNP concentration and flow rates to achieve therapeutic temperatures.	Optimizing flow and NP dose for hyperthermia therapy.	CFD simulations were not validated experimentally	Munoz et al. (2024)
Vascular modelling and biomechanics	Explores the effects of design parameters on shear stress, analyte transport, and biosensor response time.	Demonstrating CFD-guided design of integrated biosensing systems and supports fluid-aware sensor placement in vascularised chips.	CFD simulations were not validated experimentally (based on literature-derived parameters)	Wong et al. (2017)
	Uses a 3D FSI model to simulate both the pulsatile flow and mechanical deformation of tissue-engineered blood vessels under physiological conditions, providing more realistic insight than standalone CFD.	Improving biomechanical mimicry of brain vasculature.	Simulated diameter changes and average velocity Vs. Experimental microscope imaging	Wang et al. (2023)
	Simulates and validate velocity fields, shear stress conditions, and particle trajectories within bioprinted vessel geometries using HARVEY, a parallelised Lattice Boltzmann Method solver.	Identify geometry-driven attachment hotspots, informing microchannel design and flow conditions for realistic tumour cell transport and colonisation studies in GBM-on-chip systems.	Simulated velocity fields and maximum flow rates Vs. experimental particle image velocimetry (PIV) measurements	Hynes et al. (2020)
Tumour-on-a-Chip and Microenvironment Modelling	Simulates velocity profiles, shear stress distributions, and viscosity variations within the microchannel, providing insight into non-Newtonian flow behaviour, especially in regions of thrombus formation and channel bends.	Mimicking tumour vascular abnormalities and flow heterogeneities seen in tumour vasculature.	CFD velocity maps Vs. µPIV	Llenas et al. (2021)
	Simulates interstitial fluid flow (IFF) and pressure distribution across the hydrogel, optimizing the design for physiological relevance and guiding engineering of the perfusion dynamics near the vascular niche.	Guiding scaffold design and operating parameters to enable controlled replication of perivascular and peripheral microenvironments that influence tumour heterogeneity and drug resistance.	Interstitial fluid flow simulations Vs. experimentally measuring IFF *in vitro* in devices without cells, using fluorescently labelled albumin	Pun et al. (2024)
	Predicts chemical concentration gradients, flow distribution, and drug diffusion in a microfluidic brain cancer chip, informing optimal channel geometry and inlet configurations for controlled multi-drug delivery and tuneable release profiles.	Enabling precise, CFD-guided design of hydrogel-based GBM-on-chip systems for high-throughput, combinatorial drug screening.	Simulated chemical concentration distributions Vs. experimental FITC fluorescent intensities measurements	Fan et al. (2016)
	Simulates coupled oxygen diffusion, cell migration (chemotaxis), proliferation, and death in a microfluidic GBM model.	Reproducing hypoxia-driven GBM invasion patterns and enabling control of oxygenation to generate physiologically relevant necrotic and migratory structures.	Simulated alive/dead cell spatial profiles Vs. experimental fluorescence microscopy measurements	Ayensa-Jiménez et al. (2020)
	Simulates flow velocity profiles to verify laminar flow, assess velocity changes in culture chambers and mixing gradients, and confirm uniform perfusion under different inlet flow rates.	Enabling accurate drug screening and realistic tumour microenvironment replication in GBM-on-chip platforms.	CFD simulations were not validated experimentally	Steinberg et al. (2023)
Platform and device design for drug testing	Simulates 3D, time-dependent laminar flow and coupled advective–diffusive drug transport in spheroid-on-a-chip devices across >15,000 geometrical and operating parameter combinations, using parallel computing.	Providing a high-throughput computational framework to rapidly optimise microfluidic geometries and operating conditions for efficient drug delivery to tumour spheroids.	Simulated drug uptake profile Vs. quantified experimental drug uptake under two flow conditions (0.01 and 0.02 mL/h)	Kheiri et al. (2021)
	Models steady-state incompressible flow to optimise the spheroid cultivation and trapping section, ensuring uniform spheroid trapping and perfusion.	Designing microfluidic traps that can reliably isolate and perfuse GBM spheroids in-chip, enabling automated tumour spheroid handling.	CFD predictions of trapping efficiency, spheroid distribution, and flow behaviour Vs. visual observation of trapped spheroids in experiment	Panuška et al. (2024)
	Simulates velocity, shear stress, and pressure profiles across four microchannel designs by exploring effects of flow rate, channel dimensions, and micropillar spacing on local hemodynamic parameters.	Guiding geometry selection and flow parameter tuning in GBM-on-chip platforms to recreate physiologically relevant shear stress and pressure profiles, mimic heterogeneous tumour microenvironments, and enable controlled nutrient/drug delivery.	CFD simulations were not validated experimentally	Pisapia et al. (2022)
	Compares two microfluidic liver-on-a-chip designs (circular vs. elliptical chambers) using filling dynamics, velocity fields, shear stress, and air bubble entrapment.	Guiding geometry optimisation to achieve uniform flow, minimise bubble formation, and maintain physiologically relevant shear stress—key for stable perfusion and nutrient delivery in GBM-on-chip systems.	Dual simulation validation using COMSOL and ANSYS to cross-verify results Vs. experimental measurements of filling times and flow behaviour	Bakuova et al. (2023)

## 5 Critical assessment of CFD capabilities and gaps in OoC

Here, we present a critical assessment of the current literature, highlighting persistent issues such as limited experimental validation, oversimplification of biological parameters, and the absence of systematic frameworks for guiding CFD implementation in microfluidic tumour modelling. This section identifies key gaps, unresolved challenges, and methodological shortcomings that must be addressed to advance the field toward clinical and translational applications.

### 5.1 Model accuracy and validation

CFD simulations in GBM-on-chip systems often lack rigorous validation. Numerical errors from mesh resolution, discretisation schemes, and boundary condition assumptions are common, and small fabrication inconsistencies can distort microscale flows. Many studies report simulation results without benchmarking against experimental data, undermining reliability of their conclusions (García et al., 2022; Pisapia et al., 2022).

Experimental validation itself is non-trivial, requiring high-resolution techniques—such as µPIV or fluorescent tracers—not always accessible. In the absence of such data, CFD predictions—such as drug penetration or oxygen gradients—remain speculative. Improving model credibility requires better meshing practices, uncertainty quantification, and routine experimental validation. AI-based inference systems (e.g., fuzzy inference systems) can predict fluid properties at untested boundary conditions, offering data-driven confidence where direct validation is lacking (Syah et al., 2021). These practices are not yet standard across the field, but they are essential if CFD is to serve as a reliable design and analysis tool rather than a purely illustrative one. Table 4 provides a synthesis of validation steps reported for GBM-on-chip CFD models, categorised as computational or experimental, and listing their purpose, method, and relevant references to standards or literature. A detailed explanation of each validation step—including methodological guidance and relevant references—is provided in Supplementary Table S1.

**TABLE 4:** Recommended CFD validation protocol for GBM-on-chip platforms.

Validation step	Type	Purpose	Method summary
Mesh convergence	Computational	Ensure results are mesh-independent	Refine mesh incrementally, specifically at fluid–hydrogel interfaces and near-wall regions; compute Grid Convergence Index (GCI).
Time-step sensitivity	Computational	Ensure transient results are time-step independent	Reduce time step in transient simulations (e.g., for drug pulsing); check for convergence of diffusion profiles.
Code verification	Computational	Verify correct equation solving	Compare with analytical benchmarks for rectangular microchannels (Poiseuille flow) and porous flow (Darcy/Brinkman).
Solver/calculation verification	Computational	Confirm numerical convergence and stability	Monitor residuals and mass balance, especially across porous membrane boundaries.
Sensitivity analysis	Computational	Identify key input parameters and assess model robustness	Vary uncertain inputs (e.g., hydrogel permeability, PDMS diffusivity) to map impact on nutrient gradients.
Uncertainty quantification (UQ)	Computational	Estimate predictive confidence intervals	Use Monte Carlo methods to propagate experimental variance (e.g., viscosity changes) into simulation error bars.
Experimental calibration	Mixed	Fit unknown model parameters	Tune model to simple experimental cases (e.g., flow in a blank chip) before full validation
µPIV velocity field validation	Experimental	Validate CFD-predicted flow fields	Use fluorescent particles to map flow; compare line profiles or vector fields
Pressure drop validation	Experimental	Confirm bulk flow matches predictions	Measure flow rate vs. pressure drop; compare to CFD predictions
Tracer/dye gradient validation	Experimental	Validate solute transport and mixing	Introduce tracer/dye; compare experimental and simulated concentration profiles in gel regions.
Oxygen gradient validation	Experimental	Confirm hypoxia prediction matches biology	Use oxygen-sensitive microbeads or films to verify hypoxic core formation predicted by reaction–diffusion models.
Sensor calibration	Experimental	Validate in-line sensor outputs used in model comparison	Calibrate sensors (e.g., flow, pressure, O_2_) against standards; compare real-time readings to CFD predictions
Statistical comparison	Mixed	Quantify agreement between model and experiment	Apply error metrics (MAE, RMS), equivalence tests, or threshold-based validation acceptance criteria

Steps marked “Computational” are internal verification methods, while “Experimental” steps validate predictions against real-world data. “Mixed” refers to calibration stages that blend both.

### 5.2 Standardisation and reproducibility

A major obstacle in OoC design is the lack of standardisation. Differences in device geometry, cell types, matrix components, and perfusion methods make it hard to reproduce results—even within the same lab—and nearly impossible to compare findings across studies (Ustun et al., 2021; Raman et al., 2024). This issue extends to computational modelling, where groups use different software, mesh resolution, boundary conditions, and flow rates, isolating findings and complicating validation. When each platform operates by different rules, even strong findings remain isolated and difficult to verify.

Standardised protocols facilitate regulatory acceptance, especially as agencies show growing interest in OoC models as alternatives to animal testing (Pun et al., 2024). This requires devices to produce reliable, repeatable data under well-defined conditions. CFD itself contributes to this goal by offering a virtual testing ground to validate designs and conduct parametric studies that identify sensitive features and operational tolerances (Chen et al., 2020). By modelling how factors like geometry or membrane permeability affect flow behaviour and solute distribution, researchers can establish design baselines that enhance reproducibility across labs and devices. AI-driven simulation workflows can optimise CFD parameters across a range of designs, helping to identify and document sensitive design parameters, improving reproducibility.

However, real-world reproducibility depends on more than just design. Fabrication precision, material uniformity, and operator technique lie beyond CFD’s scope. Achieving high reproducibility will also require standardised fabrication protocols, rigorous quality control, and thorough reporting practices.

### 5.3 Coupling CFD with cellular and molecular data

A major gap in current approaches is the limited integration of CFD results with biological readouts. Currently, fluid flow simulations and cellular assays are often conducted in parallel without connections. Hybrid modelling frameworks that connect fluid dynamics with drug transport, cellular uptake, and therapeutic response are needed, yet remain rare. Still, progress is being made. Some researchers now advocate for *in silico* frameworks that merge multicellular behaviour with microfluidic modelling to better guide OoC design and data interpretation (Wang et al., 2025).

Achieving this integration may require an iterative workflow, where CFD predictions inform experimental setup, and the model is refined based on the experimental results. ML models trained on CFD, and experimental biology can guide OoC design by revealing relationships between flow, shear, and cell behaviour (Praharaj et al., 2024; Swamy et al., 2024). The long-term goal is to build an integrated system that correlates fluid flow, transport dynamics, and cell behaviour—an important step toward a predictable, biologically relevant OoC platform.

### 5.4 Insufficient integration of patient-specific parameters

Despite the promise of GBM-on-chip platforms for personalised medicine, most CFD models rely on some generic assumptions, e.g., standard cell lines, culture media, and idealised geometries. Achieving meaningful personalisation would require the integration of patient-specific factors, including vascular architecture, blood rheology, tumour cell phenotypes such as motility and metabolic rate, and patient-specific TME. This requires not only individualised clinical data but also the technical capacity to adapt chip design and CFD parameters accordingly. At present, both aspects remain largely underexplored. A lack of standardisation across patient-derived cell lines, along with limited access to well-characterised clinical samples, continues to undermine reproducibility in the field (Raman et al., 2024). As a result, many CFD studies remain at the prototype stage rather than advancing toward clinically relevant models.

To overcome these limitations, integrating patient-derived omics data into CFD-guided chip design could enhance personalisation. For example, a CFD simulation could be tuned to a particular patient’s tumour parameters, such as cell density, proliferation rate, tumour volume, invasion capacity, and cell phenotype, or to their MRI-derived tumour characteristics and perfusion rates. Advances in 3D printing and bioprinting might allow custom chips that mirror an individual’s tumour morphology, which CFD could then analyse. ML-driven surrogate models can be retrained with patient-specific data (e.g., tumour perfusion rates from imaging), enabling individualised CFD predictions without rerunning entire simulations (Peksen, 2024). However, achieving this will require true interdisciplinarity, bringing together clinicians (to supply patient data), experimentalists (to build patient-mimicking OoCs), and modelers (to adjust CFD frameworks for new parameters).

## 6 Discussion

**FIGURE 3:**
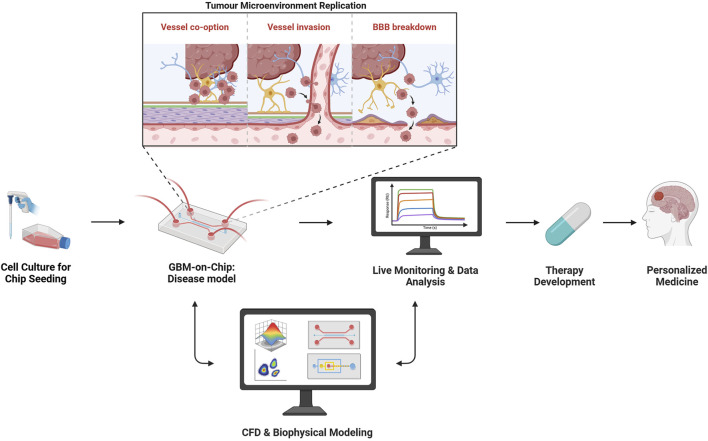
Conceptual overview of CFD integration in GBM-on-chip design. The schematic illustrates key stages, from cell culture and GBM-on-chip disease modelling to live monitoring, data analysis, and therapy development. CFD modelling supports predictive simulation of pathophysiological features and enables real-time optimisation for therapeutic applications. Created in BioRender. Taleban, H. (2025) https://BioRender.com/kp8fjqs.

Combining OoC technology with CFD represents a significant step forward in modelling the complex biology of GBM. While current GBM-on-chip systems have enhanced the ability to replicate key features of the TME, their continued dependence on empirical, trial-and-error approaches constrains both their physiological accuracy and translational potential. CFD provides a promising alternative—an engineering framework capable of rendering OoC development more systematic, quantifiable, and reproducible. As illustrated in Figure 3, CFD can be integrated throughout GBM-on-chip design, from microenvironment replication to therapy development.

This review has outlined several persistent limitations in current GBM-on-chip modelling, including the inadequate reproduction of biochemical gradients, ECM remodelling, mechanical stimuli, and BBB dynamics. CFD can address many of these challenges by simulating parameters such as mass transport, interstitial flow, shear stress, and drug diffusion with high spatial and temporal resolution. It’s crucial that CFD should not be considered as a supplementary tool but rather integrated into every stage of device design—from early prototyping to final optimisation. More importantly, two key areas emerging from the literature are highlighted: (1) the development of structured, domain-specific workflows for integrating CFD into GBM-on-chip platforms, incorporating AI-based optimisation and experimental feedback, which extend beyond previous general-purpose CFD approaches (García et al., 2022); and (2) the proposal of comprehensive validation protocols specifically tailored to CFD applications in GBM-on-chip systems. Together, these reported strategies provide a reproducible foundation for researchers aiming to move beyond descriptive modelling toward predictive, quantitative, and translatable platforms.

Looking forward, future efforts should focus on coupling CFD with patient-specific biological data—including omics, imaging, and histology—to enable truly personalised tumour modelling. Wider adoption will require collaborative initiatives to standardise workflows, improve accessibility to simulation tools, and promote interdisciplinary training that bridges engineering, computational science, and oncology. Beyond personalisation, lowering the technical barrier to CFD adoption will be essential. Cloud-based simulation platforms, standardised model templates, and AI-assisted interfaces could facilitate broader use, particularly in resource-limited academic or clinical settings. Establishing open-access libraries and robust validation protocols will also be critical to ensuring reproducibility and fostering community engagement.

In summary, while CFD offers tremendous potential to advance GBM-on-chip technologies, its integration must be intentional, methodologically rigorous, and biologically grounded. Aligning fluid dynamic precision with the complexity of tumour biology offers a path toward more predictive, clinically relevant *in vitro* models—tools capable of not only deepening our understanding of GBM but also informing therapeutic development with greater translational impact.
