# Supplementary material for: Integrating computational fluid dynamics into organ-on-chip systems: a glioblastoma-centred design and validation framework
Source: Front Bioeng Biotechnol. 2026 Jan 22;13:1716813. doi: 10.3389/fbioe.2025.1716813 (PMC12872932; doi:10.3389/fbioe.2025.1716813)
Supplement: Supplementary file 1 [file Table1.docx]

Supplementary Material

# Supplementary Tables

Table S1 Our Recommended CFD Validation Protocol for GBM-on-chip Platforms.

| **Validation Step** | **Type** | **Purpose** | **Method** | **Key References** |
| --- | --- | --- | --- | --- |
| **Mesh Convergence (Grid Independence)** | Computational | Ensure numerical solution is independent of mesh resolution (reduce discretisation error) | Run simulations with successively finer meshes; monitor key outputs (e.g. flow rate, pressure, concentration). Continue refining until changes are below a set threshold. Optionally calculate Grid Convergence Index (GCI) to quantify uncertainty | ASME V&V 20 guidance on mesh refinement, and CFD best practices (ASME, 2009; Richard G. Hills et al., 2015) |
| **Time-Step Independence (for unsteady simulations)** | Computational | Ensure transient solutions are independent of time-step sise (no integration error) | Decrease the time-step in transient CFD simulations and compare results (e.g. peak concentrations, oscillation amplitude). Choose a time-step that yields negligible change in results relative to a smaller step. | ASME V&V 20 transient V&V (ASME, 2009) |
| **Code Verification** | Computational | Verify that the simulation code solves governing equations correctly (no programming errors) | Test the CFD code against analytical solutions or benchmark cases. For example, compare simulated Poiseuille flow in a simple channel to analytical velocity profile; check mass conservation in a closed-loop simulation. Use published benchmark data such as (Hariharan et al., 2017) for verification | ASME V&V 20-2009, CFD code verification (ASME, 2009); FDA benchmark studies (FDA, 2016) |
| **Solver/Calculation Verification** | Computational | Ensure the specific simulation setup is numerically accurate (solver settings, convergence) | Check that residuals are minimised, and solution iterative convergence is achieved. Perform localised refinements or alternative solver schemes to see if results remain consistent. Confirm no significant numerical artifacts (e.g. oscillations) in results. | ASME V&V 20 standard (ASME, 2009) |
| **Sensitivity Analysis** | Computational | Identify which input parameters most influence model outputs (robustness check) | Vary key inputs one-factor-at-a-time or via design of experiments (e.g. ±10–20% changes in viscosity, permeability, cell uptake rates). Observe changes in outputs (flow rate, gradients). Map sensitivities to determine which parameters require accurate measurement and which have minor effect | ASME V&V 40 emphasises sensitivity studies for risk assessment (ASME, 2018); FDA guidance on model risk informs depth of sensitivity needed (FDA, 2023) |
| **Uncertainty Quantification (UQ)** | Computational | Quantify the impact of input and model uncertainties on simulation predictions (provide confidence bounds) | Define distributions or ranges for uncertain inputs (e.g. diffusion coefficient, inlet flow variability). Use Monte Carlo simulations or analytic uncertainty propagation to compute confidence intervals for outputs. For example, report that drug concentration at tumour core is X ± Y%. Document any biases. If uncertainties remain, record and note whether they likely make predictions conservative or non-conservative. | ASME V&V 40 framework for risk-informed UQ (ASME, 2018); OECD PBK Model Guideline on documenting uncertainties (OECD, 2021) |
| **Experimental Calibration (if needed)** | Both | Calibrate model parameters to match experimental baseline (before validation) | If certain parameters are unknown or tuneable (e.g. wall drag coefficient, cell permeability), perform calibration experiments. Fit the CFD model to a simple case (e.g. straight-channel pressure drop, or diffusion in a blank chip without cells) to estimate these parameters. Use the calibrated values in subsequent simulations. | OECD guidance on model calibration and reporting (OECD, 2021) |
| **µPIV Velocity Field Validation** | Experimental | Validate velocity field and shear predictions in microchannels or porous regions | Seed the chip’s fluid with fluorescent micro-particles. Use micro-particle image velocimetry to capture velocity vectors in regions of interest (e.g. channel, around tumour spheroid). Compare vectors and velocity profiles to CFD predictions – qualitatively (flow patterns) and quantitatively (line profiles of velocity). Ideally, compute error metrics (e.g. mean difference) between experimental and simulated velocities. | Example: (Rito Pereira et al., 2022) micro-PIV vs CFD in micro-canals showed close agreement; µPIV widely used for Lab-on-chip validation (Silva et al., 2008) |
| **Pressure Drop / Flow Rate Validation** | Experimental | Verify overall flow behaviour (circuit analogy) in device against model | Measure the pressure drop across the device at known flow rates using a manometer or pressure sensor. Alternatively, impose a pressure drop and measure resulting flow rate. Compare to CFD-predicted pressure-flow relationship. This checks the bulk hydraulic resistance of the chip (important for overall perfusion). | Standard fluid mechanics (e.g., Hagen–Poiseuille) for baseline; (FDA, 2016) benchmark nozzle or microfluidic network models; Ensures no major leaks or mis-modelled resistances in CFD |
| **Tracer/Dye** **Visualisation of Gradients** | Experimental | Validate species transport and mixing predictions (concentration fields) | Introduce a coloured or fluorescent tracer into the device to establish a concentration gradient or mixing interface (e.g. a dye in one inlet of a Y-channel). Capture images or intensity profiles over time. Compare the measured concentration profiles or front propagation speed with CFD simulation of the tracer. For steady gradients (e.g. in a gradient generator), compare the profile shape (linear, exponential, etc.) between experiment and simulation. | (Marturano-Kruik et al., 2018) validated CFD-predicted flow velocities using fluorescent microspheres in bone-on-chip to; (Tonon et al., 2019) used ruthenium dye to validate O₂ gradient shape |
| **Oxygen Gradient & Concentration Validation** | Experimental | Confirm CFD-predicted oxygen (or nutrient) distributions and identify hypoxic zones correctly | Use oxygen-sensitive fluorescent sensors (e.g. ruthenium-based dye or optical sensor spots) in the chip. Calibrate the sensor (21% O₂ and 0% O₂ references). Measure oxygen levels at various positions under operating conditions. Compare to CFD-predicted O₂ values at those positions. Additionally, use hypoxia markers (e.g. pimonidazole staining in cells, or HIF-1α immunostaining) after an experiment to see if regions predicted to be hypoxic indeed show hypoxia biologically. This provides qualitative validation that the oxygen depletion predicted by the model has biological effects in those regions. | (Ando et al., 2017) embedded O₂ sensor particles in a tumour chip, showing sensor readings matched simulation and correlated with pimonidazole staining; (Guidance Document on Good In Vitro Method Practices (GIVIMP), 2018) suggests using analytical measurements to validate gradients in microphysiological systems |
| **Sensor Integration and Calibration** | Experimental | Validate model using on-chip sensor data (flow, pressure, etc.), ensuring sensor accuracy | Calibrate on-chip sensors (flow sensors, pressure transducers, oxygen probes) with known standards (e.g. known flow rates from a pump, known gas concentrations). During chip operation, record sensor outputs (e.g. real-time flow rate or oxygen level) and compare against CFD-predicted values at the same sensor location/conditions. Use calibrated correction factors if needed. This real-time validation can also track if the model holds over time (e.g. does predicted flow remain stable and equal to measured flow over hours). | Microfluidic oxygen sensor calibrated at 0% and 21% O₂ to validate gradient formation (Rexius-Hall et al., 2017) |
| **Statistical Comparison and Acceptance Criteria** | Both | Quantitatively assess model vs. experiment agreement and decide if model is “validated” for purpose | Apply statistical tests or error metrics to the validation data. For instance, use the student’s t-test or equivalence tests to see if simulation and experiment differ significantly, accounting for uncertainty. Define acceptance criteria (e.g. “CFD and experiment must agree within 10% for key outputs”). If criteria are met, model is validated for that condition (context of use). If not, identify whether discrepancies are due to model error or experimental uncertainty, and iterate. A threshold-based approach can be used: if differences are small relative to a critical threshold (e.g. a safety limit of shear stress), the model may be acceptable | FDA’s “threshold-based” validation approach for medical CFD models (Hariharan et al., 2017b); ASME V&V 40 provides a structured risk-based acceptance approach (Muhammad Jamil and Nathan Müller, n.d.); Standard metrics: mean absolute error, RMS error, Bland-Altman analysis for biofluid devices |

# References

Ando, Y., Ta, H. P., Yen, D. P., Lee, S.-S., Raola, S., and Shen, K. (2017). A Microdevice Platform Recapitulating Hypoxic Tumor Microenvironments. *Sci Rep* 7, 15233. doi: 10.1038/s41598-017-15583-3

ASME (2009). Standard for Verification and Validation in Computational Fluid Dynamics and Heat Transfer (ASME V&V 20-2009). New York.

ASME (2018). Assessing Credibility of Computational Modelling through Verification and Validation: Application to Medical Devices (ASME V&V 40-2018). New York.

FDA (2016). Reporting of Computational Modeling Studies in Medical Device Submissions. Silver Spring, MD. Available at: https://www.fda.gov/media/87586/download? (Accessed September 30, 2025).

FDA (2023). Assessing the Credibility of Computational Modeling and Simulation in Medical Device Submissions. Silver Spring, MD. Available at: https://www.fda.gov/media/154985/download? (Accessed September 30, 2025).

Guidance Document on Good In Vitro Method Practices (GIVIMP) (2018). Paris.

Hariharan, P., D’Souza, G. A., Horner, M., Morrison, T. M., Malinauskas, R. A., and Myers, M. R. (2017a). Use of the FDA nozzle model to illustrate validation techniques in computational fluid dynamics (CFD) simulations. *PLoS One* 12, e0178749. doi: 10.1371/journal.pone.0178749

Hariharan, P., D’Souza, G. A., Horner, M., Morrison, T. M., Malinauskas, R. A., and Myers, M. R. (2017b). Use of the FDA nozzle model to illustrate validation techniques in computational fluid dynamics (CFD) simulations. *PLoS One* 12, e0178749. doi: 10.1371/journal.pone.0178749

Marturano-Kruik, A., Nava, M. M., Yeager, K., Chramiec, A., Hao, L., Robinson, S., et al. (2018). Human bone perivascular niche-on-a-chip for studying metastatic colonization. *Proceedings of the National Academy of Sciences* 115, 1256–1261. doi: 10.1073/pnas.1714282115

Muhammad Jamil, and Nathan Müller (n.d.). Computational Modelling & Simulation in Medical Device Development. *Starfish Medical*. Available at: https://starfishmedical.com/resource/computational-modelling-simulation-medical-device-development/? (Accessed September 30, 2025).

OECD (2021). Guidance Document on the Characterisation, Validation and Reporting of Physiologically Based Kinetic (PBK) Models for Regulatory Purposes. Paris. Available at: https://www.oecd.org/content/dam/oecd/en/publications/reports/2021/02/guidance-document-on-the-characterisation-validation-and-reporting-of-physiologically-based-kinetic-pbk-models-for-regulatory-purposes_670da2f4/d0de241f-en.pdf (Accessed September 30, 2025).

Rexius-Hall, M. L., Rehman, J., and Eddington, D. T. (2017). A microfluidic oxygen gradient demonstrates differential activation of the hypoxia-regulated transcription factors HIF-1α and HIF-2α. *Integrative Biology* 9, 742–750. doi: 10.1039/C7IB00099E

Richard G. Hills, David C. Maniaci, and Jonathan W. Naughton (2015). V&V Framework (SAND2015-7455). Albuquerque, NM & Livermore, CA.

Rito Pereira, M., Silva, G., Semiao, V., Silverio, V., Martins, J. N. R., Pascoal‐Faria, P., et al. (2022). Experimental validation of a computational fluid dynamics model using micro‐particle image velocimetry of the irrigation flow in confluent canals. *Int Endod J* 55, 1394–1403. doi: 10.1111/iej.13827

Silva, G., Leal, N., and Semiao, V. (2008). Micro-PIV and CFD characterization of flows in a microchannel: Velocity profiles, surface roughness and Poiseuille numbers. *Int J Heat Fluid Flow* 29, 1211–1220. doi: 10.1016/j.ijheatfluidflow.2008.03.013

Tonon, F., Giobbe, G. G., Zambon, A., Luni, C., Gagliano, O., Floreani, A., et al. (2019). In vitro metabolic zonation through oxygen gradient on a chip. *Sci Rep* 9, 13557. doi: 10.1038/s41598-019-49412-6
